# A Novel Fabrication of Single Electron Transistor from Patterned Gold Nanoparticle Array Template-Prepared by Polystyrene Nanospheres

**DOI:** 10.3390/nano12183102

**Published:** 2022-09-07

**Authors:** Jingyue Fang, Xinxing Li, Wenke Xie, Kehui Sun

**Affiliations:** 1 School of Physics and Electronics, Central South University, Changsha 410073, China; 2 Key Laboratory of Nanodevices, Suzhou Institute of Nano-Tech and Nano-Bionics, CAS, Suzhou 215213, China

**Keywords:** single electron transistor, gold nanoparticle, self-assemble, Coulomb blockade

## Abstract

In this paper, polystyrene microspheres were firstly prepared by seeded emulsion polymerization, and the uniform monolayer of polystyrene microspheres was prepared on the substrate by the dipping method. Then, polystyrene monolayer film was used as a mask and a low dimensional array structure of gold was prepared by bottom-up self-assembly process. After that, the method of solution etching and annealing was used, and the gold nanoparticle array was post-processed. As a result, gold nanoparticles were recrystallized, with an average diameter of about 50 nm. Subsequently, the semiconductor process was adopted, with focused ion beams induced deposition and electron beam evaporation, and single electron transistors were fabricated, based on self-assembled gold nanoparticles. Finally, the devices were fixed in a liquid helium cryostat and Coulomb blockade was observed at 320 mK. It is a novel fabrication of a single electron transistor based on gold nanoparticle array template and prepared with polystyrene nanospheres.

## 1. Introduction

As early as 1951, Cornellis Gorter, who is a member of the Kamerlingh Onnes laboratory in the Netherlands, reported Coulomb blocking, studying the single electron phenomenon [1]. Nanoparticles have long been prepared by physical or chemical methods. These nanoparticles served as a Coulomb Island and a single-electron transistor could be prepared by this bottom-up method, to study the single electron phenomenon. In 1995, Chen et al. prepared AuPd nanoparticles with dimensions of 2–3 nm. The single electron transistor constructed exhibited a significant Coulomb blocking effect at 77 K and nonvolatile voltammetry features even at room temperature [2]. In 1996, David L. Klein et al. used a size~5.8 nm Au nanoparticle and CdSe nanoparticles to construct a single electron transistor, and a clear Coulomb step curve was observed at a temperature of 77 K [3]. In 1997, Toshihiko Sato et al. used the molecular self-assembly method to locate Au quantum dots with a diameter of ~10 nm on silicon substrates and prepared a single electron transistor. Coulomb steps and Coulomb oscillations were observed at 4.2 K [4]. In 2010, Hidehiro Yamaguchi et al. used an oligothiophene pentamer to assemble Au quantum dots between nanoelectrodes and measured the differential conductance image at 12 K, the image showed a typical Coulomb diamond, indicating that the device was a single electron transistor [5].

There are three key technical problems in the preparation of existing single-electron transistors: controllable preparation of a small-sized Coulomb Island; controlled positioning assembly of the Coulomb Island; precise control of the size of tunneling barrier between the Coulomb Island and electrode. This is related to the device’s operating temperature and its performance consistency. Therefore, researchers have long been eager to develop a method that can precisely control the size and location of the Coulomb Island, and control the size of the barrier between the Coulomb Island and the electrode to greatly facilitate the preparation and application of single-electron transistors. For example, by fabricating a narrow gap between the source and drain electrodes, the access resistance is reduced and the number of dots involved in the SET operation also diminishes [6]. Willing S. et al. adopted the method of self-assembly of colloidal metal nanoparticle strips, and, since the size of metal nanoparticles and the tunnel barrier width are adjustable, the transistor showed controllable characteristics [7]. In this paper, we propose a method for preparing single electron transistors. The size of Au particles can be controlled by controlling the particle size in a PS microsphere template, the coating time, wet etching and annealing. The thickness of the barrier layer can be precisely controlled by controllable thin film preparation methods, such as double beam deposition or atomic layer deposition. In the double beam system, the in-situ device preparation and the assembly and positioning of the Coulomb island and electrode for the regular periodically arranged Au particle array also becomes simpler. The electron tunneling characteristics of single electron transistors with quantum dots as the core structure have been the frontier and hotspot of the research. Based on this characteristic, it is possible to develop highly sensitive single photon detection devices.

## 2. Materials and Methods

### 2.1. Preparation of Polystyrene Microspheres

Monodispersed polystyrene (PS) microspheres were prepared by seed emulsion polymerization. First, the styrene was washed with sodium hydroxide, dried with anhydrous calcium chloride, and then heated to about 50 to 60 °C in a vacuum distillation apparatus to complete the purification of the reagent. Then, the preparation of monodispersed PS microspheres started. Nitrogen gas was introduced into a three-necked round bottom flask in the presence of a reflux device and equipped with a mechanical stirrer. Then, deionized water and alcohol were added, the mixture was stirred and rapidly added to the styrene, reacting for 8 h to obtain a seed emulsion. The effect of the temperature and the stability of the stirring speed were relatively large. Alternatively, it could be stirred for a while, and the initiator reaction added after the system stabilized.

### 2.2. Preparation of Polystyrene Microspheres Monolayer

**Figure 1:**
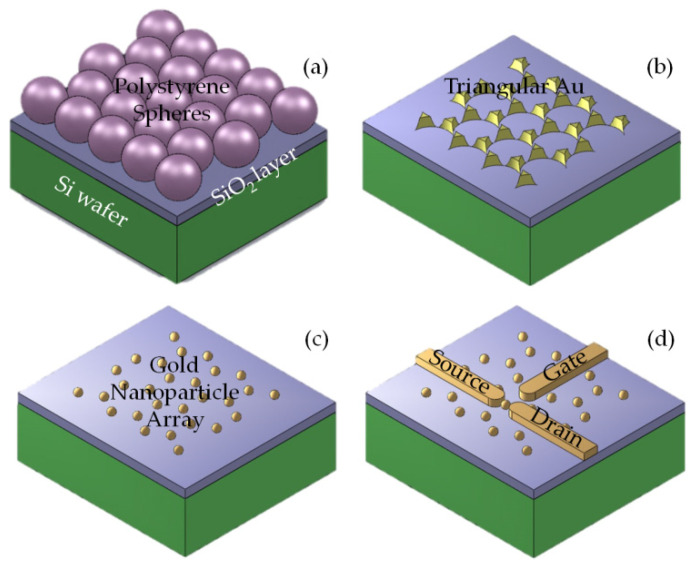
Schematic diagram of the process for SET preparation. (**a**) The PS microsphere monolayer was prepared on Si wafer; (**b**) Triangular gold array was prepared by the PS microsphere template; (**c**) Gold nanoparticle array was prepared by means of solution etching and annealing; (**d**) After 6 nm-thick-SiO_2_ was deposited on the gold nanoparticle array, typical electrodes were prepared.

After preparation of the PS microsphere emulsion, the substrate was subjected to hydrophilic treatment, and then the PS microsphere template was prepared by the pulling assembly [8,9]. PS microsphere mixed liquor was applied to the washed and dried quartz substrate, and it was observed that the white mixed liquor spread evenly on the substrate. A certain amount of distilled water was then trickled into the culture dish and the substrate sank slowly into the water. The white nanosphere suspension spread evenly on the liquid surface and formed a large area of the film floating on the liquid surface. After a period of time, 2% of the sodium dodecyl sulfate solution was dropped on the liquid surface to change the surface tension of the liquid surface. The liquid surface dropped from the center to the surrounding sudden diffusion so that the film was more closely arranged. When the liquid surface was stable, the substrate fixed on the moving rail was moved down at a certain speed from the blank surface until the substrate sunk into the water. A small amount of sodium dodecyl sulfate solution was added dropwise to “drive” the selected film onto the substrate until the liquid level was stabilized and pulled at a uniform and slow rate to transfer the film onto the substrate completely dry. Then the PS microsphere monolayer film template, shown in Figure 1a, was obtained.

### 2.3. Preparation of Gold Nanoparticle Arrays

Furthermore, we used monolayer polystyrene microsphere templates as masks to prepare gold nanoparticle arrays [10]. The metal was deposited on the substrate on which the PS microsphere template was assembled by means of electron beam evaporation coating. After deposition, the mixture was ultrasonically cleaned in an anhydrous ethanol and chloroform mixed solution (volume ratio 1:10) for 5 min to wash away the mask plate, then the metal nano-array structure, shown in Figure 1b, was obtained. The metal nanostructure was post-treated by solution etching or annealing to obtain a recrystallized nanoparticle array (Figure 1c). The temperature was 800 °C, the atmosphere was Ar:H_2_ = 2:1 (volume ratio), the flow rate was Ar/H_2_, 500/250 sccm, the temperature control process was 60 min uniform from room temperature to 800 °C, and then constant temperature for 1 h, and then natural cool down.

### 2.4. Preparation of Single Electron Transistors Based on Gold Nanoparticles

On the samples of gold nanoparticle arrays that were obtained, a gold nanoparticle was selected as the Coulomb Island, and SiO_2_ was deposited with a focused ion beam to a thickness of 6.0 nm in a dual beam system (FEI Helios Nanolab 600i). The ion beam source voltage and current were set at 30 kV and 40 pA, respectively. It could also be achieved by using ALD. Then, using the Coulomb Island as the center, deposition of platinum for the preparation of electrodes was induced. The organ metallic precursor (CH_3_)_3_Pt(CpCH_3_) resulted in platinum incorporated carbon-rich samples. To remove the C and Ga elements in the sediments, the platinum metal content was increased. The samples were placed in H_2_O_2_:H_2_SO_4_ = 1:1 solution for 60 s to remove the C and other substances. Then the samples were rinsed with deionized water and dried with N_2_. The samples were then allowed to stand in a high-temperature combustion tube furnace at a rate of 20 °C/min and annealed at 500 °C for 30 min under an O_2_ atmosphere (flux 30 sccm) [11]. Finally, the device was bonded to get the single-electron transistor device (Figure 1d).

## 3. Results and Discussion

### 3.1. Template of PS Microsphere Monolayer

**Figure 2:**
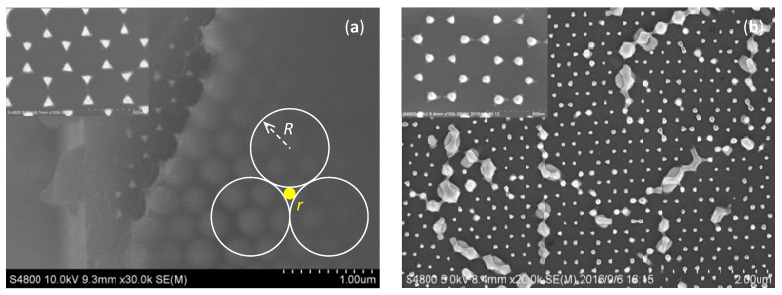
SEM images of (**a**) polystyrene sphere mono-film covered with a 300 nm-thick gold film, and (**b**) gold nanoparticle array after post treatment. The left up inset of (**a**) shows triangular gold array. The right down inset of (**a**) shows the geometrical relationship of polystyrene sphere and gold nanoparticle. The inset of (**b**) shows that the gold nanoparticle formed after annealing is well crystallized with a diameter of about 60 nm.

After the preparation of the PS microsphere emulsion, we first carried out the hydrophilic treatment of the substrate, and then prepared the PS microsphere template by the pulling assembly method. Then, electrons were deposited on the PS microsphere template by electron beam evaporation coating, and the results are shown in Figure 2a. The mask of coated PS spheres was lifted off with a mixed solution of absolute ethanol and chloroform to obtain a gold nanoparticle array structure, as shown in the illustration of Figure 2a. The nanoparticle array obtained was in the form of a hexagonal array of triangular structures with side lengths of 90 mm. We further controlled the shape and size of the gold nanoparticles by means of post-treatment, such as wet etching and annealing. According to the geometrical relationship (inset of Figure 2a), many triangular spaces implanted among the six-angle dense pile. The circles in the triangular spaces touched the neighboring polystyrene spheres. The relationship between the radius of the circles and the polystyrene spheres could be written as √3(*r* + *R*)/2 = *R*. So, the radius of the circle *r* was equal to 30.9 nm when the radius of the polystyrene sphere *R* was 200 nm. If we wanted to get gold nanoparticles with a size less than 10 nm, the diameter of the polystyrene sphere would need to be less than 65 nm. As shown in Figure 2b, after 800 °C annealing, the triangular nano gold was converted into granular form. Since the gold nanoparticles were annealed at high temperatures, the particles could release stress to recrystallize and, thus, became spherical.

**Figure 3:**
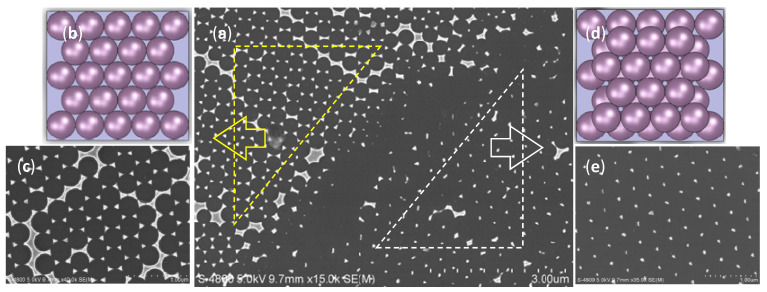
(**a**) SEM image of gold nanoparticles arrays template-fabricated by monolayer and bilayer PS microspheres array template on the same substrate; (**b**) Monolayer PS microspheres array template; (**c**) Gold nanoparticles arrays fabricated by the template as (**b**); (**d**) Bilayer PS microspheres array template; (**e**) Gold nanoparticles arrays fabricated by the template as (**d**).

In the preparation of PS template, we obtained the monolayer template. However, in some local areas there were also cases of bilayer films. As shown in Figure 3, the shape and geometry of the gold nanoparticles array fabricated by the bilayer PS microsphere array template were quite different. The gold nanoparticle array template prepared by monolayer PS microsphere array template had a triangular honeycomb layout (Figure 3c). In contrast, the gold nanoparticle array template prepared by bilayer PS microsphere array template had a point array triangle layout (Figure 3e).

### 3.2. Device Preparation

**Figure 4:**
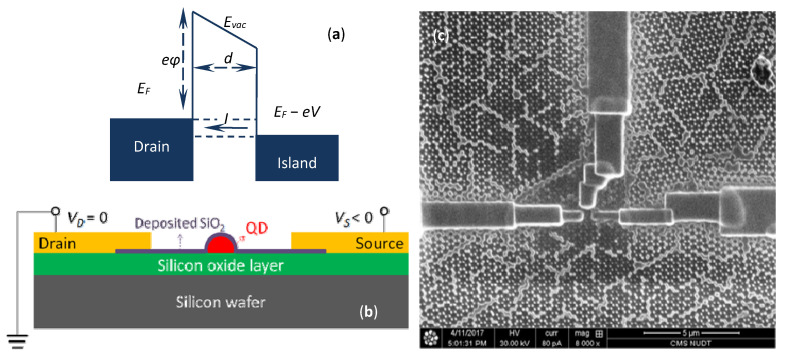
(**a**) Band diagram of electrode-dielectric-gold nanoparticle junction; (**b**) Schematic diagram of the side view of the SET device; (**c**) SEM image of the top view of the SET device.

As shown in Figure 4a, there was an insulating layer of silicon oxide on gold nanoparticles, which was deposited by focused ion beams induced deposition (FIBID). Successively, platinum was deposited for electrodes preparation by the method of focused electron beam induced deposition (FEBID) and FIBID. Then, taking post-processing measures, such as annealing, to complete the preparation of the device, a single electron transistor device was the result, as shown in Figure 4c. The compositional changes of the wet etching and thermally annealed FIBID and FEBID materials were investigated before in [11]. EDX results are shown in Table 1. The results implied that the wet etching method was not effective. However, the platinum purity of the calcined sample was higher. It was possible that carbon and gallium reacted with oxygen during the annealing, and carbon dioxide molecules escaped with the gas flow. Therefore, the platinum content would be relatively increased and the conductivity would be enhanced. In addition, the annealing treatment was an alternative method to avoid current leakage.

The tunneling resistance if electrons go from the drain to the gold nanoparticle can be calculated. The corresponding band diagram of electrode–dielectric–gold nanoparticle junction under a bias *V* is shown in Figure 4b, where *d* is the channel length, *eφ* is the work function, *E_F_* is the Fermi energy and *E_vac_* is the vacuum level. When a battery lowers the energy levels in the gold nanoparticle with respect to the drain contact, and maintains them at distinct electrochemical potentials separated by *eV*, the numbers of electrons can be given by different Fermi functions: *f*(*E*, *E_F_* − *eV*, *T*) and *f*(*E*, *E_F_*, *T*). Supposing that the tunneling probability of electrons inflow from the drain to the gold nanoparticle is the same as the one of electrons outflow in the opposite direction, we can write the current through this junction as follows [12]:

(1) $$\begin{array}{l} I=-\frac{2e}{h}\sum_{n=1}^{N}\int_{-\infty }^{+\infty }\left(f\left(E,E_{F}-eV,T\right)-f\left(E,E_{F},T\right)\right)T_{n}\left(E\right)dE\\ =\frac{2e^{2}V}{hk_{B}T}\sum_{n=1}^{N}\int_{0}^{E_{F}}\frac{\exp\left(\frac{E-E_{F}}{k_{B}T}\right)}{{\left(1+\exp\left(\frac{E-E_{F}}{k_{B}T}\right)\right)}^{2}}\exp\left(-\frac{4\sqrt{2m}}{3e\varepsilon \hbar }{\left(e\varphi -\left(E-E_{F}\right)\right)}^{\frac{3}{2}}\right)dE \end{array}$$

where *e* and *m* are the electron charge and mass, respectively; *h* is the Planck constant (*ħ* = *h*/2π); *N* is the total number of sub-bands below Fermi energy; *E* is the electron energy; *k_B_* is the Boltzmann constant; *T* is the Kelvin temperature and *ε* is the electric field intensity. *T_n_* is the tunneling probability of electrons in the energy level of *n*, in the form of Fowler-Nordheim tunneling formulation.

The resistance of the channel determines the current that flows between the drain and the gold nanoparticle when a voltage *V_ds_* is applied. According to Equation (1), an equivalent tunneling resistance *R* with varying thicknesses of potential barrier length *d* could be calculated. As Table 2 shows, the tunneling resistance between the metallica electrode and a gold nanoparticle at 320 mK and 2 K were calculated. The following parameters were used in the calculations: Fermi energy *E_F_* = 5.51 eV (gold); modified work function *eφ* = 4.1 eV (interface of gold-SiO_2_); cross section dimension of the drain 50 × 50 nm^2^. The results showed that when the thickness of the silicon oxide layer was in a thickness range of 3.0–6.5 nm, the magnitude of the tunneling resistance was in the range of 10^4^–10^13^ Ω. This was a proper value for the electron tunneling, while larger resistance would lead to much lower tunneling probability and feebler tunneling current.

### 3.3. Device Test

**Figure 5:**
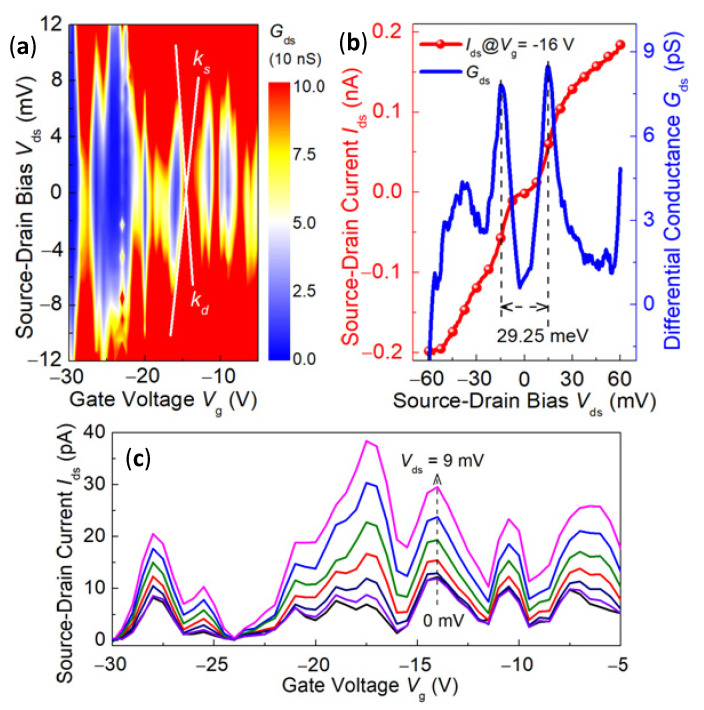
(**a**) Source–drain differential conductance as a function of source drain bias *V*_ds_ and gate bias *V*_g_ at *T* = 320 mK; (**b**) The measured source–drain current curve and its corresponding differential conductance when gate voltage was set at −16 V; (**c**) The measured source-drain current curve as a function of gate bias *V*_g_, when source drain bias *V*_ds_ was set at different valuea, from bottom to top of 0 mV to 9 mV.

Coulomb blockade oscillations can be seen clearly in the electrical characteristics of the SET measured at 320 mK. The dependence of the Coulomb blockade on *V*_ds_ and *V*_g_ was investigated. Differential conductance versus *V*_g_ and *V*_ds_ is shown in Figure 5a. The blue diamond indicated that the electrons had a Coulomb blockade and the number of electrons on the gold nanoparticle island kept constant. However, the red region indicated that single electron tunneling occurred, which is a typical characteristic of a single electron transistor. The slopes of the two bevel edges of the prismatic when gate voltage was equal to −16 V were *k*_s_ = *k*_d_ = 7.2 × 10^−3^, respectively. The Coulomb diamond had symmetry along the *V*_ds_ = 0 direction, which indicated that the source and drain tunneling junctions of the single electron transistor were symmetrical, and if the junction capacitance of the source and drain was different, the Coulomb diamond would tilt. Figure 5b shows the source–drain current curve and its corresponding differential conductance when gate voltage was set at −16 V. The Coulomb blockade effect could be clearly seen, both in the *I*_ds_-*V*_ds_ and *G*_ds_-*V*_ds_ curves. The length of the low conductive region Δ*V*_ds_ was about 29.25 mV and the charging energy of the Coulomb Island was estimated to be 29.25 meV, which was much larger than the thermal energy of 138.1 × 10^−4^ meV at 320 mK. The capacitance *C*_d_ of 2.74 aF was achieved with a zero-biased resistance of ~1.05 GΩ. The corresponding thickness of the silicon oxide barrier was calculated to be about 6.3 nm. It was in agreement with the device preparation, as before. There was also a capacitance between the Coulomb Island and the gate, and the source of either. According to the relationships *k*_s_ = *C*_g_/(*C*_g_ + *C*_s_) and *k*_d_ = *C*_g_/*C*_d_, *C*_g_ = 0.02 aF and *C*_s_ = 2.72 aF, the total capacitance was *C*_∑_= *C*_s_ + *C*_d_ + *C*_g_ = 5.48 aF. If we assumed that the capacitance of a spherical Coulomb Island with a radius r was given by *C*_∑_ = 4π*ε_r_ε*_0_*r*, the dot diameter was calculated to be 24.6 nm with the dielectric constant of SiO_2_ *ε_r_* = 4. It was consistent with the range of island diameter observed in the SEM image of GNPs.

When the source drain voltage did not change, the source leakage current changed with the change of the gate voltage, which is called the Coulomb oscillation effect. As shown in Figure 5c, with increase of the source drain voltage, the values of the Coulomb oscillation peaks increased. This was because, as the source drain voltage increased, the number of levels that fell into the source drain potential window increased, and each time the number of electrons tunneling through the source–island–drain increased; thus. the tunneling current became larger.

**Figure 6:**
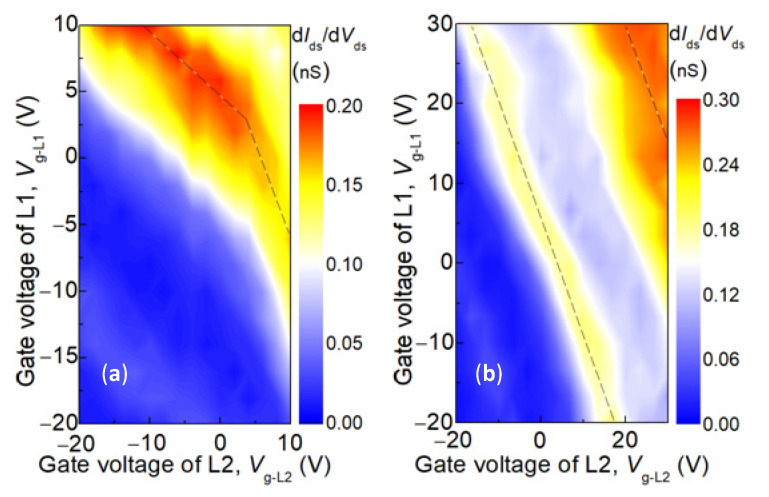
Double quantum dot systems are controlled by gate pressure with different coupling intensities. (**a**) There is capacitive coupling between quantum dots, one electron on one quantum dot changes the electrostatic energy of the other quantum dot; (**b**) The coupling capacitance evolves to the main capacitance, and the separation of the triple point reaches the maximum.

Considering the double quantum dot systems in series, their electrochemical potential was independently controlled by their respective side gate electrodes. When there was a capacitance coupling between the quantum dots, adding an electron to one of the quantum dots changed the electrostatic energy of the other quantum dot. Moreover, the gate pressure of the quantum dots was generally capacitive coupled to the quantum dots. The charge stability diagram shows a hexagonal honeycomb. Figure 6a shows a local part of the honeycomb lattice. The point of the honeycomb lattice is called the triple point. At a triple point, the energy of different charge states is degenerate. The distance between the triple points is determined by the coupling strength between the quantum dots. When the coupling between the two quantum dots was very strong, as shown in Figure 6b, the separation of the three state points reached the maximum, and the behavior of the double quantum dots became like that of only one quantum dot.

## 4. Conclusions

In this paper, we used the seed emulsion method to prepare polystyrene microspheres of specific size, and then, by using the dip coating method, a PS microsphere monolayer film was prepared, and it was used as a template for metal evaporation. After removing the template of PS microspheres, the periodic array of gold nanoparticles was obtained. The size of the gold nanoparticles could be controlled and the crystallinity of the particles improved by the method of solution etching and annealing, helping to improve its electrical performance. Based on this, 6 nm thick silicon oxide was deposited on the gold nanoparticles using double beam equipment, and deposition of the source, drain and gate, subsequently. Due to the presence of impurities, such as C and Ga, in the material obtained by the focused ion beam induced deposition, it was necessary to improve the conductivity of the material by high temperature annealing in an oxygen atmosphere. Finally, the differential conductance test was carried out using the liquid helium cryostat. While changing the gate voltage and sweeping the source drain voltage, under the interaction effect of the Coulomb step effect and the Coulomb oscillation effect, the Coulomb diamond of the single electron transistor conductance was observed. A single electron transistor was made by a novel bottom-up self-assembly method. On the basis of the platform, single electron phenomena, such as single photon assisted tunneling, can be further studied.

## Figures and Tables

**Table 1:** The atomic concentrations of the dual beam-induced resultants.

Element	FIBID			FEBID		
	Deposition	Wet Etching	Calcination	Deposition	Wet Etching	Calcination
C (K)	73.74	70.72	24.23	91.68	90.76	/
O (K)	/	/	34.70	/	/	75.90
Ga (L)	10.16	9.39	14.61	3.73	2.15	/
Pt (M)	16.10	19.88	26.46	4.59	7.09	24.10

**Table 2:** Equivalent resistance of barrier with different thickness.

*d* (nm)		3.0	3.5	4.0	4.5	5.0	5.5	6.0	6.5
***R*** **(100 kΩ)**	**@2 K**	0.64	10.89	185.23	3.15 × 10^3^	5.36 × 10^4^	9.13 × 10^5^	1.55 × 10^7^	2.64 × 10^8^
	**@320 mK**	0.10	1.74	29.64	5.04 × 10^2^	8.58 × 10^3^	1.46 × 10^5^	2.49 × 10^6^	4.23 × 10^7^

## Data Availability

All data, models, and code generated or used during the study appear in the submitted article. They are available from the corresponding author by request (J.F.).
